# lncRNA ZFAS1 Positively Facilitates Endothelial Ferroptosis via miR-7-5p/ACSL4 Axis in Diabetic Retinopathy

**DOI:** 10.1155/2022/9004738

**Published:** 2022-08-31

**Authors:** Yu Liu, Zhengyu Zhang, Jing Yang, Jingfan Wang, Yan Wu, Rongrong Zhu, Qinghuai Liu, Ping Xie

**Affiliations:** ^1^Department of Ophthalmology, The First Affiliated Hospital of Nanjing Medical University, Nanjing 210029, China; ^2^Department of Ophthalmology, The Affiliated Hospital of Nantong University, Nanjing 226000, China; ^3^Nanjing University, Nanjing 210029, China

## Abstract

Accumulating evidence has suggested the significant role of long noncoding RNAs (lncRNA) in regulating ferroptosis, while its regulatory mechanism in diabetic retinopathy (DR) remains unelucidated. In this work, we first demonstrated that lncRNA zinc finger antisense 1 (ZFAS1) is upregulated in high glucose-cultured human retinal endothelial cells (hRECs) and ZFAS1 inhibition attenuated high glucose- (HG-) induced ferroptosis, which was evidenced by cell viability, total iron and ferrous iron levels, reactive oxygen species (ROS) level, and Glutathione Peroxidase 4 (GP_X_4) expression detection. Mechanistically, we validated that ZFAS1 may act as a competing endogenous RNA by competitively binding with microRNA-7-5p (miR-7-5p) and modulating the expression of its downstream molecule acyl-CoA synthetase long-chain family member 4 (ACSL4), which is now identified as a classic driver gene of ferroptosis process. In conclusion, our results demonstrate that HG-induced ZFAS1 elevation activates ferroptosis in hRECs and the ZFAS1/miR-7-5p/ACSL4 axis may serve as a therapeutic target for endothelial dysfunction in DR.

## 1. Introduction

Diabetic retinopathy (DR) is a complication of diabetes mellitus which seriously affects visual health. The number of diabetes mellitus patients was estimated to be 9.3% (463 million people) globally in 2019 [1]. Approximately 30% of patients with diabetes mellitus deteriorate into DR, and the mechanism and treatment of DR have always been the focus of medical research [2–4]. It has been well documented that microvascular endothelial cells are sensitive targets of hyperglycemia [5, 6]. In nonproliferative stage of DR, excess microvascular cell death was observed to be involved in the subsequent diabetic neuropathy by cutting down blood supplies to nervous system [7–10]. Given the initial role of microvascular cell death in diabetic retinopathy progression, efforts should be made to prevent or slow down the retinal microvascular cell loss process.

Apoptosis is known to be a major contributor to endothelial cell death [11, 12]. However, several studies have pointed out that apoptosis itself cannot explain all the endothelial loss processes, making exploring new forms of cell death urgently needed [13–15]. Ferroptosis is a newly identified nonapoptosis cell death, characterized by lethal accumulation of intracellular iron and iron-induced lipid reactive oxygen species (ROS) [16, 17]. The overaccumulation of ROS production leads to an oxidative stress response in cells that causes unfold or misfold proteins and cytoplasmic swelling and eventually to cell death [18–20]. Accumulating evidence reported that ferroptosis is involved in diverse biological processes and diseases, including immune disease, cancer, and neurodegenerative diseases [21–24]. Intriguingly, ferroptosis processes share many similar features with diabetes-induced endothelial dysfunction, both characterized by reactive oxygen species (ROS) accumulation and enhanced oxidative stress [25, 26]. Recently, Luo et al. reported that HG and interleukin-1 beta can induce human umbilical vein endothelial cells ferroptosis, which indicates that ferroptosis is involved in endothelial dysfunction [27]. However, the regulatory mechanism underlying the ferroptosis-mediated endothelial dysfunction has yet been elucidated.

A growing number of evidences have suggested that lncRNAs are involved in multiple biological processes through a number of different signaling pathways [28, 29]. lncRNA ZFAS1 is a lncRNA on chromosome 20 and has been reported to play either oncogenic or tumor suppressor role in different cancers [30–33], while its role in endothelial dysfunction requires further study. In our current study, we discovered that the expression level of lncRNA ZFAS1 was upregulated under hyperglycemia in both RNA sequencing dataset and in cultured hRECs. Further, our results revealed that ZFAS1 exerts its efforts by acting as a miR-7-5p sponge to regulate the ACSL4 expression. Recently, Zhuang et al. reported that upregulation of miR-7-5p promotes ferroptosis by regulating levels of transferrin receptor, uptake of iron, and production of lipid reactive oxygen species in cardiomyocyte [34]. Another group of researchers found that knockdown of miR-7-5p in malignant cells results in the downregulation of the iron storage gene expression such as ferritin as well as the upregulation of the ferroptosis marker gene expression [35]. ACSL4, a lipid metabolizing enzyme required for ferroptosis and a driver gene of ferroptosis process, catalyzes the linkage of long-chain poly-unsaturated fatty acids to coenzyme A and preferentially utilizes long PUFAs for functions in phospholipid biosynthesis [36, 37]. In this work, we provide new insights into the molecular function of ZFAS1 in DR, supporting the notion that ZFAS1 may serve as a therapeutic target for DR treatment. Moreover, we proved the potential diagnostic and therapeutic application of ZFAS1/miR-7-5p/ACSL4 axis in DR treatment.

## 2. Methods

### 2.1. Data Collection and Bioinformatics Analysis

GEO dataset was downloaded from GEO database (http://www.ncbi.nlm.nih.gov/geo). The GSE94019 dataset contained CD31^+^ endothelial cells isolated from nine fibrovascular membrane samples from patients with proliferative DR and four control retinal samples without diabetes diagnosis. The “limma” package in R was employed (|log2(FC)| = 0.5 and *P* < 0.05) to process and identify the differentially expressed lncRNA.

### 2.2. Cell Culture

Primary hRECs were purchased from PromoCell (C-12200, Heidelberg, Germany), and all of the experiments were performed using 2-5 passages of hRECs. hRECs were grown in complete endothelial culture medium ECM (ScienCell, 1001) containing 1% endothelial cell growth supplement (ScienCell, 1052), 5% fetal bovine serum (Gibco, A3160802), and 1% penicillin/streptomycin solution (Gibco, 15140-122) at 37°C in a humidified atmosphere of 5% carbon dioxide. Cell culture plates and centrifuge tubes were purchased from NEST Biotechnology Co. Ltd. (Wuxi, China). For high glucose cells, experiments indicated that the amount of D-glucose (MCE, HY-B0389) was added directly in ECM media to obtain a final concentration of 10, 15, 20, or 30 mM, and a hypertonic group (24.5 mM mannitol and 5.5 mM glucose) was added to exclude hyperosmolarity effects. To detect the role of ferroptotic signals in HG-induced endothelial dysfunction, hRECs were treated with 10 *μ*M apoptosis inhibitor tauroursodeoxycholic acid (TUDCA) (MCE, 35807-85-3), 10 *μ*M necrosis inhibitor necrostatin-1 (MCE, 4311-88-0), 10 *μ*M ferroptosis inhibitor ferrostatin-1 (Fer-1) (MCE, HY-100579), and 10 *μ*M pyroptosis inhibitor tetraethylthiuram disulfide (TETD) (MCE, 97-77-8) for 48 h.

### 2.3. Plasmid and shRNA Transfection

hRECs were plated in a 6-well plate with the density of 10^5^ cells per well. The ZFAS1 shRNA (Sh-ZFAS1), miR-7-5p mimics/inhibitor, and blank plasmids were purchased from GenePharma (Shanghai, China). Transfection of miRNA mimics/inhibitor and shRNAs was conducted using Lipo 3000 transfection agent (Invitrogen) according to the manufacturer's instruction. The shRNA sequences used in this study were presented in Supplementary Table 1. The pCDNA3.1-ZFAS1 plasmids were generated by inserting the ZFAS1 coding sequences into pCDNA3.1 empty vector (Invitrogen). After 18 h of starvation, the cells were transfected, and 72 hours later, the efficacies of this shRNA were validated by RT-qPCR before being used in subsequent experiments.

### 2.4. Cell Viability

Cell viability was performed using trypan blue staining method as previously described [38]. Briefly, cells were seeded in a 6-well plate at densities of 10^5^ per well with 2 ml medium. After attachment, hRECs were transfected or treated with various plasmids and cultured for 48 h. 10 *μ*l cell suspension was mixed up with 10 *μ*l 0.1% trypan blue solution (Keygen, KGY015) and pipetted into a blood cell counting plate. To assess the cell viability in each experimental group, three randomly selected fields were counted manually by two experienced evaluators under a stereomicroscope.

### 2.5. Measurement of Lipid Peroxidation Levels

C11-BODIPY assay was used to assess lipid peroxidation according to the manufacturer's instruction. Cells were seeded into 6-well dishes at a concentration of 10^6^ cells per well. After treatment, cells were stained with BODIPY 581/591 C11 (Thermo Fisher Scientific, D3861) for 30 min at 37°C in the dark and then washed twice using PBS and then detected at an emission wavelength of 510 nm and an excitation wavelength of 488 nm.

### 2.6. Measurement of Total and Ferrous Iron

The measurement of total and ferrous iron level in cell samples was conducted using Iron Assay Kit (Sigma, Cat. # MAK025) [39]. Cells were plated at 1 × 10^5^ cells/well in a six-well plate. 48 h later, cell samples were collected using 12,000 × *g* centrifugation for 10 min at 4°C. In each group, 5 *μ*l of assay buffer was first added in 50 *μ*l sedimentation to measure total iron, and 5 *μ*l of iron reducer buffer was then added to convert Fe^3+^ to Fe^2+^, before being adjusted to a final volume of 100 *μ*l per well in a 96-well plate with assay buffer. The plate was gently shaken for 30 min at room temperature. After, 100 *μ*l iron probe buffer was added in each well, and the system was shaken for another 60 min at room temperature. The plate was protected from light during the whole procedure, and the absorbance was measured at 593 nm on fluorescence microplate reader.

### 2.7. Dual-Luciferase Reporter Assay

The dual-luciferase reporter assay was performed to validate the interaction between lncRNA ZFAS1 and miR-7-5p, or between miR-7-5p and ACSL4. Briefly, wild-type and mutated 3′-untranslated region (3′-UTR) regions of ZFAS1 luciferase reporter gene vectors (named ZFAS1-WT and ZFAS1-MUT, respectively) were designed and synthesized by Guangzhou RiboBio Co., Ltd., China. The cells were transfected with miR-7-5p mimic or NC-mimic and the ZFAS1-WT or ZFAS1-MUT for 48 h. Cells were then lysed to detect luciferase activity using the dual-luciferase reporter assay system (Promega, Madison, MI, USA) according the manufacturer's instruction. Likewise, cells were transfected with miR-7-5p mimic or miR-NC and the ACSL4-WT or ACSL4-MUT for 48 h, before subsequent luciferase activity was detected.

### 2.8. Cellular Fractionation

To determine the subcellular localization of ZFAS1, hRECs were lysed on ice and fractionated using the Cell Fractionation Kit (Abcam, ab109719) following the manufacturer's instructions. Nuclear and cytoplasmic fractions were then analyzed by western blotting. GAPDH and U6 were utilized as cytoplasmic and nuclear markers, respectively.

### 2.9. RNA Fluorescence In Situ Hybridization

Specific fluorescence-conjugated probes for ZFAS1 were designed and synthesized by Life Technologies (Shanghai). The signals of the probe were detected by FISH Kit (GenePharma, Shanghai) according to the manufacturer's instructions. Nuclei were stained with DAPI. The images were monitored and captured using a Leica confocal microscopy (Leica Microsystems, Mannheim, Germany).

### 2.10. Western Blotting

Western blotting was performed as previously described [40]. After 72 hours of shRNA transfection, hRECs were washed once with ice-cold PBS and then resuspended in RIPA buffer (HY-K1001, MCE, USA) with protease and phosphatase inhibitors. The samples were then lysed by a constant vortex for 30 min at 4°C, after which the protein concentration of the supernatant was determined by the Pierce BCA protein assay kit (#23225, Thermo Fisher, USA). A total of 30 *μ*g protein samples were loaded on 10% sodium dodecyl sulfate-polyacrylamide gel electrophoresis gels, transferred to PVDF membrane (Merck–Millipore, USA), and blocked with 5% skim milk in TBST. The target proteins were immunodetected using GP_X_4 (1 : 1000, ab125066, Abcam) and GAPDH (1 : 1000, A2228, Sigma) antibodies following overnight incubation at 4°C. The protein bands were detected by Chemistar substrate (Tanon, Nanjing). Bio-Rad Quantity One software was employed to quantify the intensity of the protein bands.

### 2.11. Quantitative Real-Time PCR

Total RNA from the cells was extracted using the TRIzol method (Invitrogen, 10296010) following the manufacturer's instructions. Total RNA (1.5 *μ*g) was reverse transcribed to cDNA by random hexamers and SuperScript IV (Invitrogen). Power SYBRGreen Mix (Thermo Fisher, 4367659) and StepOnePlus real-time PCR system (Applied Biosystems) were then used to quantify relative RNA ratio. Samples were biologically triplicated for mean ± SEM. The primer sequences for RT-qPCR are listed in Supplementary Table 1, and GAPDH and U6 were used as internal references.

### 2.12. Diabetic Retinopathy Model

A total of 48 C57BL/6J male mice at six weeks old were purchased and randomly assigned to four groups: controls, diabetic, diabetic with sh-ZFAS1 transfection, and diabetic with sh-ZFAS1 transfection and miR-7-5p inhibitor (*n* = 12 in each group). Streptozotocin (STZ) (Sigma-Aldrich, 55 mg/kg) was injected intraperitoneally for 5 consecutive days to induce hyperglycemia, and controls were injected intraperitoneally with an equivalent volume of sodium citrate buffer [41–43]. All mice accepted intravitreal injection of plasmids (Sh-ZFAS1 or miR-7-5p inhibitor) or vehicles six weeks post the first STZ injection.

### 2.13. Isolation of CD31^+^ RECs Using Flow Sorting

RECs were isolated from the retinae in four experimental groups using the flow sorting method as described before [44, 45]. In each experimental group, mice were euthanized, and their retinas were carefully enucleated for subsequent analysis. Retinae were dissociated using 1 mg/ml type II collagenase (Worthington, cat. #LS004176), washed with DPBS three times, and then decanted through the 40-*μ*m strainer. After that, cells were labeled with isotype control or anti-CD31 antibody (Cat. No. 563607, BD Bioscience), kept in the dark, and incubated on ice for 30 min. After positive selection for CD31, cells were washed three times and subsequently sorted on a FACS Aria (BD Biosciences), and the CD31^+^ RECs were finally obtained.

### 2.14. Immunostaining Assay

To characterize the primary hRECs as shown in Supplementary Figure 1, cells were fixed in 4% paraformaldehyde (PFA) for 15 min and permeabilized with 0.5% Triton X-100 and 1% BSA for 15 min at room temperature. Cells were blocked with 5% BSA for 1 h and incubated overnight at 4°C with the anti-CD31primary antibodies (1 *μ*g/ml, ab281583, Abcam). Cells were counterstained with DAPI (Southern Bio, 0100-20), mounted using LSM-880 confocal fluorescence microscope (Carl Zeiss, Jena, Germany). For in vivo experiments, the eyes in each group (*n* = 6) were enucleated carefully and processed for indirect immunofluorescence in whole-mount or cross-section as previously described [46]. For cryosections, the eyes (*n* = 3 retinae from 3 mice) were fixed in 4% PFA at room temperature for 15 min. The frozen samples were then sliced transversely (6 *μ*m) at -20°C. For retinal flat-mounts, the eyes (*n* = 3 eyes from 3 mice) were fixed in 4% PFA at room temperature for 15 min, and the retinae were dissected out as cups. Both cryosections and retinal cups were blocked with PBS containing 0.5% Triton-X100 and 5% BSA at 4°C overnight and included with the anti-CD31 and anti-GP_X_4 (1 : 100, ab125066, Abcam) primary antibodies.

### 2.15. Statistical Analysis

The data presented are representative of at least three independent experiments and are presented as mean ± SEM. Statistical analysis was performed in GraphPad. *P* values were determined by ANOVA with Tukey HSD post hoc test, and *P* value less than 0.05 was considered statistically significant. Pearson's correlation analysis analyzed the correlation between the ZFAS1 and miR-7-5p level in hRECs. Significance between samples is denoted as ^∗^*P* < 0.05 and ^∗∗^*P* < 0.01.

## 3. Results

### 3.1. ZFAS1 Is Upregulated in hRECs under High Glucose

**Figure 1:**
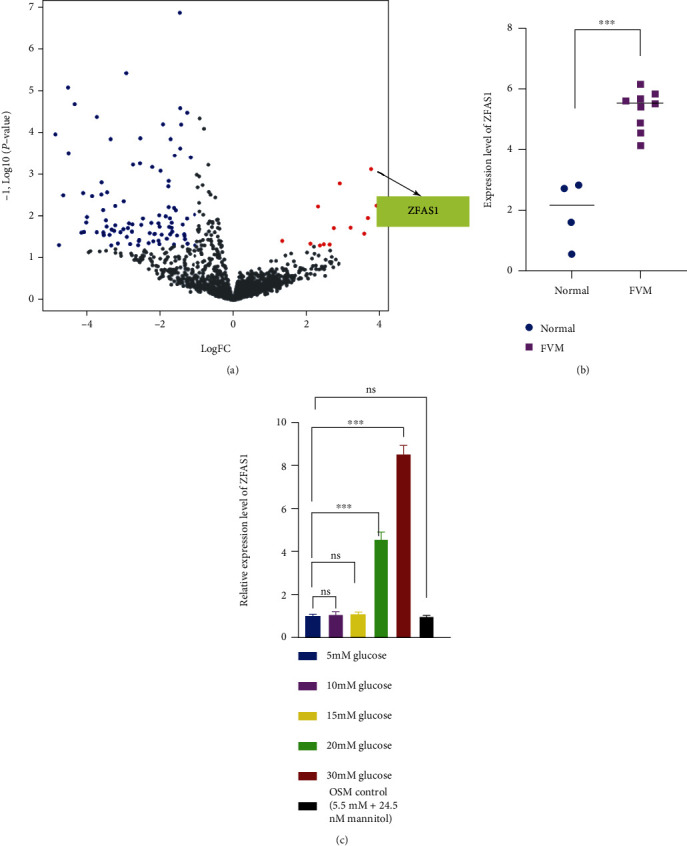
ZFAS1 is upregulated in hRECs under HG and in FVM tissues. (a) The volcano map of differentially expressed lncRNAs in GEO: GSE94019. (b) The expression level of lncRNA ZFAS1 in endothelial cells isolated from FVM samples and healthy control retinal samples. (c) RT-PCR was performed to detect the ZFAS1 expression cultured in LG (5.5 mM), HG (10, 15, 20, and 30 mM), or hypertonic control group (5.5 mM glucose and 24.5 mM mannitol) medium for 48 h. *n* = 5 replicates. Data are presented as ± SEM. ^∗∗∗^*P* < 0.001.

A total of 108 dysregulated lncRNAs were identified between the CD31^+^ endothelial cells isolated from nine fibrovascular membrane (FVM) samples and four control retinal samples without diabetes diagnosis using a differential gene expression analysis (Figure 1(a) and Supplementary Table 2). Among the significantly upregulated lncRNAs, ZFAS1 was chosen for further investigation for its previously reported role in promoting ferroptosis [47]. Given that hyperglycemia is now regarded as the primary cause of DR by activating subsequent interconnecting biochemical pathways, the expression levels of ZFAS1 were detected in low and high glucose-cultured hRECs. In consistent with RNA sequencing result from GSE94019 dataset (Figure 1(b)), RT-qPCR results validated that HG (25 mM and 30 mM) stimulation for 48 h generated high level of ZFAS1 expression compared with that under low glucose (LG) (5.5 mM) (Figure 1(c)).

### 3.2. ZFAS1 Knockdown Alleviates High Glucose-Induced Ferroptosis

**Figure 2:**
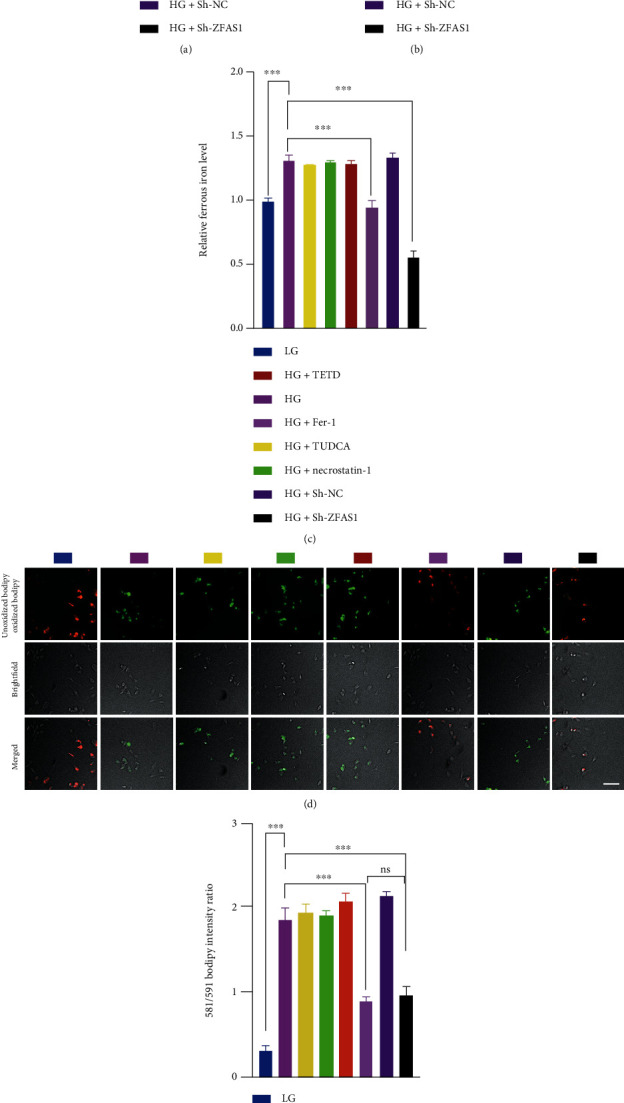
Inhibition of ZFAS1 rescued HG-induced ferroptosis in hRECs. (a) Both apoptosis inhibitor TUDCA and ferroptosis inhibitor Fer-1 administration rescued the downregulated cell viability in HG group. Compared to HG control, there is no difference in total (b) or ferrous iron level (c), lipid hydroperoxide accumulation (d and e), nor GPx4 expression level (f) after 10 *μ*M apoptosis inhibitor TUDCA, necrosis inhibitor necrostatin-1, pyroptosis inhibitor TETD treatment for 48 h, while transfection of Sh-ZFAS1 remarkably lightened the above ferroptosis phenotypes. *n* = 5 replicates. *P* > 0.05. 10 *μ*M Fer-1 treatment for 48 h notably ameliorated the HG-induced ferroptosis-related phenotypes indicated above. *n* = 5 replicates. Data are presented as ± SEM. Ns: no significant; ^∗∗^*P* < 0.01 and ^∗∗∗^*P* < 0.001. Scale bar, 50 *μ*m.

It has been reported that lncRNA ZFAS1 can promote ferroptosis and finally accelerates the progression of pulmonary fibrosis; one can expect that ZFAS1 may be associated with hyperglycemia-induced endothelial dysfunction, which is also associated with ferroptosis processes according to previous studies [27]. In line with Luo et al.'s study, cell viability of hRECs was compromised, and the iron accumulation was aggravated under HG for 48 h (Figures 2(a)–2(c)). C11-BODIPY probe was employed to assess the lipid peroxidation level as described before [48]. An increase of oxidated to unoxidated C11 ratio was observed after HG treatment, suggesting the ability of HG condition to induce lipid peroxidation-related ferroptosis (Figures 2(d) and 2(e)). As the sole enzyme in mammalian cells to eliminate lipid ROS, the expression level of GP_X_4 determines cell fate upon ferroptotic signals [49]. As shown in Figure 2(f), HG treatment dramatically downregulated the protein abundance of ferroptosis-associated gene GP_X_4. Moreover, all the above changes could be reversed by ferroptosis inhibitor ferrostatin-1 but not by TUDCA, necrostatin-1, and TETD (inhibitors of apoptosis, necrosis, and pyroptosis, respectively), except for the cell viability downregulation was rescued by ferrostatin-1 and TUDCA treatment, indicating the crucial role of ferroptosis process in HG-induced endothelial dysfunction. In addition, quantification of the GP_X_4 intensity showed that the GP_X_4 protein level was higher in HG+shZFAS1 group compared to HG+Fer-1 group, while the lipid peroxidation level in these two groups showed no significant difference. This asynchronism indicated that there may exist other mechanisms involved in the Fer-1 preventing the accumulation of lipid peroxidation products, apart from its reported role in maintaining GP_X_4 expression [50, 51]. It is worth noting that both quantifications of lipid ROS and GP_X_4 expression here cannot be used as the determinants to assess ferroptosis activity separately. The precise mechanism under this asynchronism requires further investigations.

To further determine whether the upregulation of ZFAS1 is involved in HG-induced ferroptosis, Sh-ZFAS1 as well as its scramble control (Sh-NC) was transfected into hRECs. Cell viability assay revealed that depletion of ZFAS1 significantly restored the cell viability repressed by HG. Moreover, ZFAS1 silencing resulted in the deactivation of ferroptosis, as reflected by significantly diminished lipid peroxidation level and relatively high levels of GP_X_4 expression. Altogether, our results demonstrate that ZFAS1 knockdown alleviates high glucose-induced ferroptosis in hRECs.

### 3.3. ZFAS1 Served as miR-7-5p Sponge

**Figure 3:**
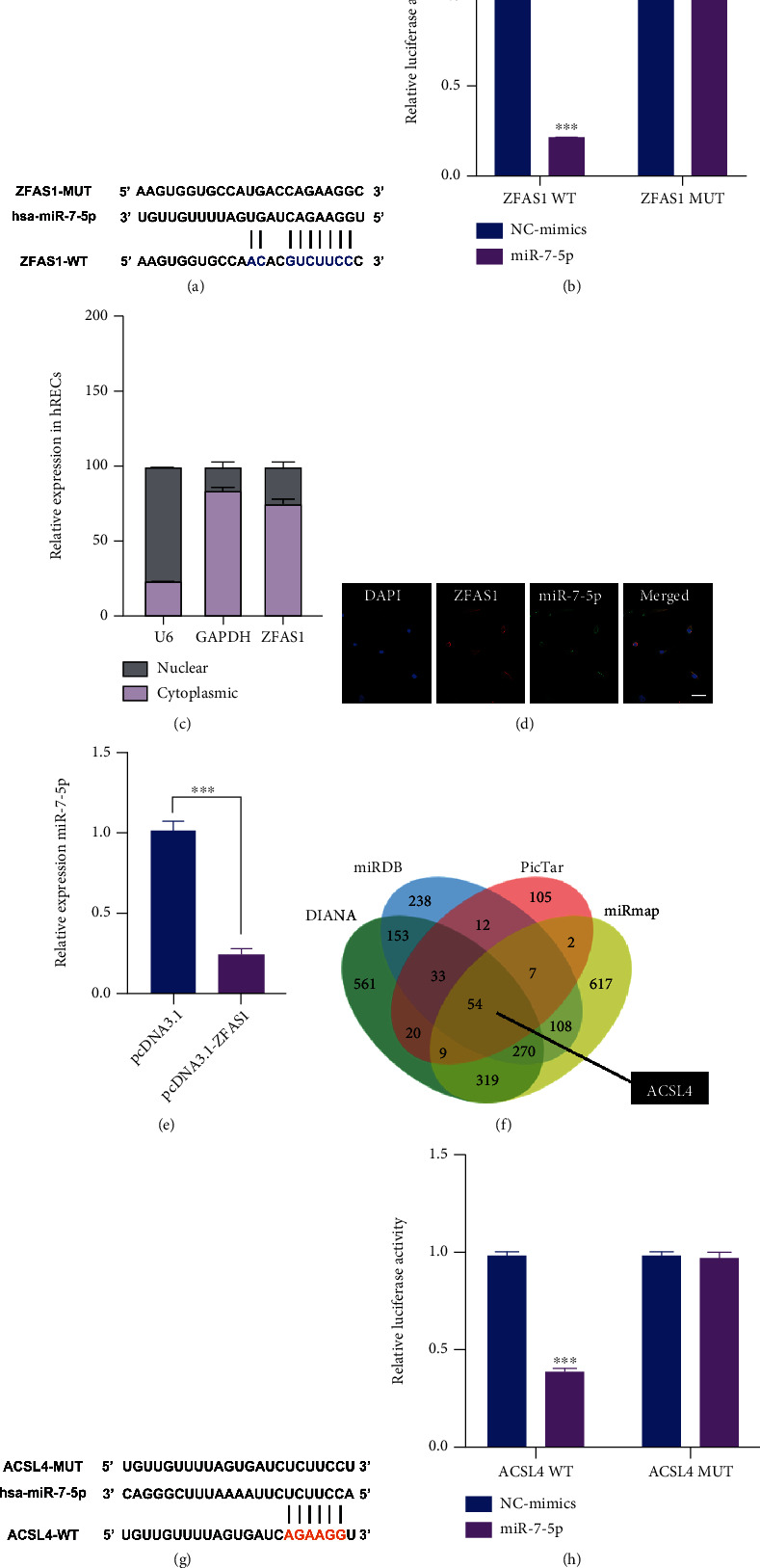
ZFAS1 regulates ACSL4 expression by directly interacting with miR-7-5p. (a) The binding sites of ZFAS1 with miR-7-5p as predicted by starBase. (b) Transfection of miR-7-5p mimics remarkably attenuated the luciferase activity of ZFAS1-WT compared with negative control groups. (c) RT-qPCR was employed to detect the expression of lncRNA ZFAS1 in the cytoplasm and nucleus of hRECs. GAPDH and U6 served as cytoplasmic and nuclear markers, respectively. *n* = 5 in each group. (d) Colocalization between ZFAS1 (labeled in red) and miR-7-5p (labeled in green) was observed by RNA FISH in hRECs. Scale bar, 25 *μ*m. (e) hRECs were transfected with empty vector pcDNA3.1 or pcDNA3.1-ZFAS1 for 48 h, and the expression level of miR-7-5p was validated using RT-qPCR. miR-39-3p served as an exogenous normalization. *n* = 5 in each group. (f) Five different datasets were utilized to predict the downstream molecule of miR-7-5p. (g) The binding sites of miR-7-5p with ACSL4 as predicted by starBase. All data are presented as ± SEM. ^∗∗∗^*P* < 0.001. (h) miR-7-5p mimics remarkably attenuated the luciferase activity of ACSL4-WT compared with negative control groups.

Evidence has confirmed that lncRNAs act as competing endogenous RNAs (ceRNAs) by competing for binding to microRNAs. To obtain insight into the regulatory mechanism for the observed ferroptosis promoting phenotypes of ZFAS1, we further tested the downstream signal. A total of 69 miRNAs were identified to interact with ZFAS1 using starBase database (Supplementary Table 3), among which miR-7-5p was chosen for further evaluation for its reported role in regulating ferroptosis in various cell types, including cardiomyocyte, malignant HeLa cells, and clinically relevant radioresistant cancer cells [34, 35, 52]. Dual-luciferase reports assay was performed to verify the potential combination between ZFAS1 and miR-7-5p (Figures 3(a) and 3(b)). Subsequently, the subcellular distribution of ZFAS1 in hRECs was validated using subcellular fractionation and fluorescence in situ hybridization (FISH) assays. As shown in Figures 3(c) and 3(d), subcellular fractionation and FISH results both revealed that ZFAS1 was mainly localized in the cytoplasm of hRECs. Moreover, we also observed that the expression of miR-7-5p in hRECs was significantly repressed after ZFAS1 overexpression, indicating the negative regulation of ZFAS1 on miR-7-5p (Figure 3(e)).

Further, the potential target genes of miR-7-5p were predicted using predictive datasets miRDB, DIANA, miRmap, and PicTar (http://mirdb.org/; http://carolina.imis.athena-innovation.gr/diana_tools/web/index.php; https://mirmap.ezlab.org/; https://pictar.mdc-berlin.de/) (Figures 3(f) and 3(g) and Supplementary Table 4). ACSL4 was chosen for subsequent experiments for its previously reported roles in inducing ferroptosis in retinal pigmental epithelial cells in DR. Luciferase activity assay showed that the luciferase activity of ACSL4-WT was inhibited by miR-7-5p mimics, while ACSL4-MUT not affected (Figure 3(h)).

### 3.4. ZFAS1 Promoted Ferroptosis through miR-7-5p/ACSL4 Axis

**Figure 4:**
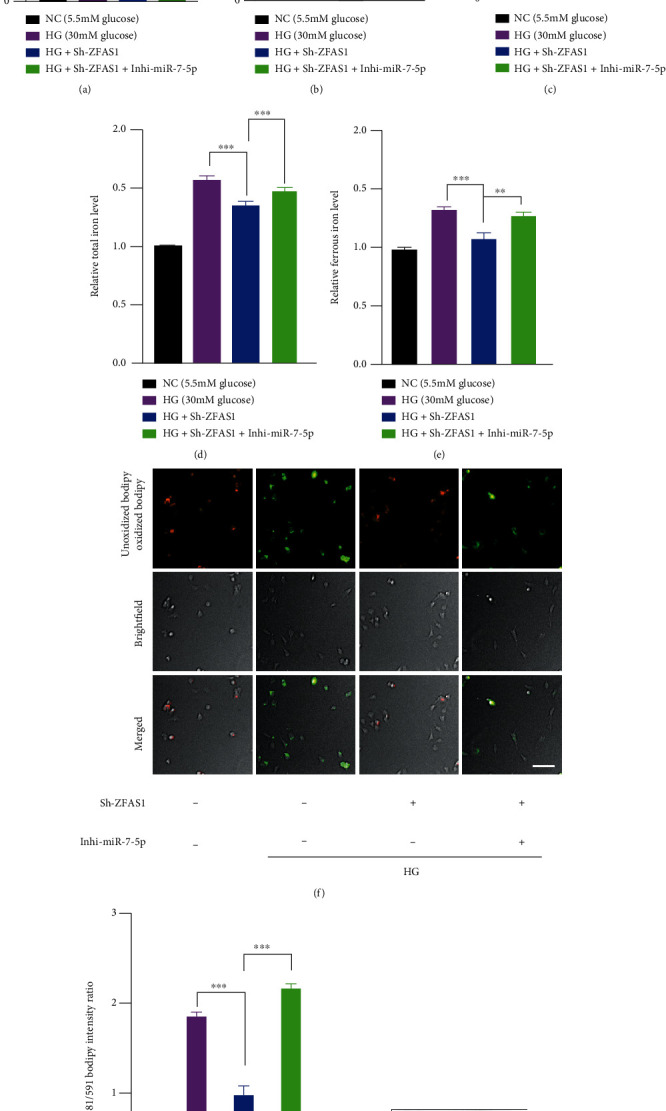
The effect of ZFAS1 silencing on preventing ferroptosis was abolished by dual knockdown of ZFAS1 and miR-7-5p. RT-qPCR results demonstrated the miR-7-5p (a) and ACSL4 expression level (b). GAPDH and miR-39-3p served as endogenous and exogenous normalization for ACSL4 and miR-7-5p detection, respectively. *n* = 5 replicates. Data are presented as ± SEM. ^∗∗∗^*P* < 0.001. (c–g) hRECs were transfected with 50 *μ*M Sh-ZFAS1 or together with 50 *μ*M miR-7-5p inhibition, and the cell viability (c), total (d), ferrous iron level (e), and lipid hydroperoxide accumulation (f and g) were assessed 72 hours after the transfection. *n* = 5 replicates. Data are presented as ± SEM. ^∗∗∗^*P* < 0.001. Scale bar, 50 *μ*m. (h) Analysis of GPx4 and ACSL4 expression level.

To investigate whether ZFAS1 exerts its role through miR-7-5p/ACSL4 axis, hRECs were transfected with Sh-ZFAS1 or together with miR-7-5p inhibition (Inhi-miR-7-5p). As shown in Figures 4(a) and 4(b), RT-qPCR showed that Sh-ZFAS1 transfection restored the repressed expression level of ACSL4 under HG, whereas the Inhi-miR-7-5p downregulated its expression by approximately six times. We further compared the ferroptosis-related phenotypes in the above experimental groups. Transfection of Sh-ZFAS1 resulted in a relatively higher cell viability and less total and ferrous iron level in hRECs compared with their negative controls, while miR-7-5p depletion brought them all to a basal level (Figures 4(c)–4(e)). Collectively, C11-BODIPY assay showed that lipid peroxidation level was significantly elevated, and the GP_X_4 expression was downregulated following knockdown of the miR-7-5p, both indicating the activation of ferroptosis processes (Figures 4(f)–4(g)). Western blotting result revealed that the HG-brought ACSL4 overexpression was restrained by ZFAS1 silencing, and this effect was largely reversed by miR-7-5p inhibition (Figure 4(h)). Of note, we overexpress GP_X_4 to further detect whether it can influence the miR-7-5p/ACLS4 axis. As validated in Supplementary Figure 2, the expression level of miR-7-5p as well as ACLS4 was not detectably altered after GP_X_4 overexpression, indicating that GP_X_4 is more likely a downstream molecule of ZFAS1/miR-7-5p/ACSL4 axis. Taken together, these results imply that ZFAS1 may modulate ferroptosis-mediated endothelial dysfunction through ACSL4, a well-recognized promotor of ferroptosis, by sponging miR-7-5p [53–55].

### 3.5. ZFAS1/miR-7-5p/ACSL4 Axis Modulates Endothelial Ferroptosis in Diabetic Mice Retinae

**Figure 5:**
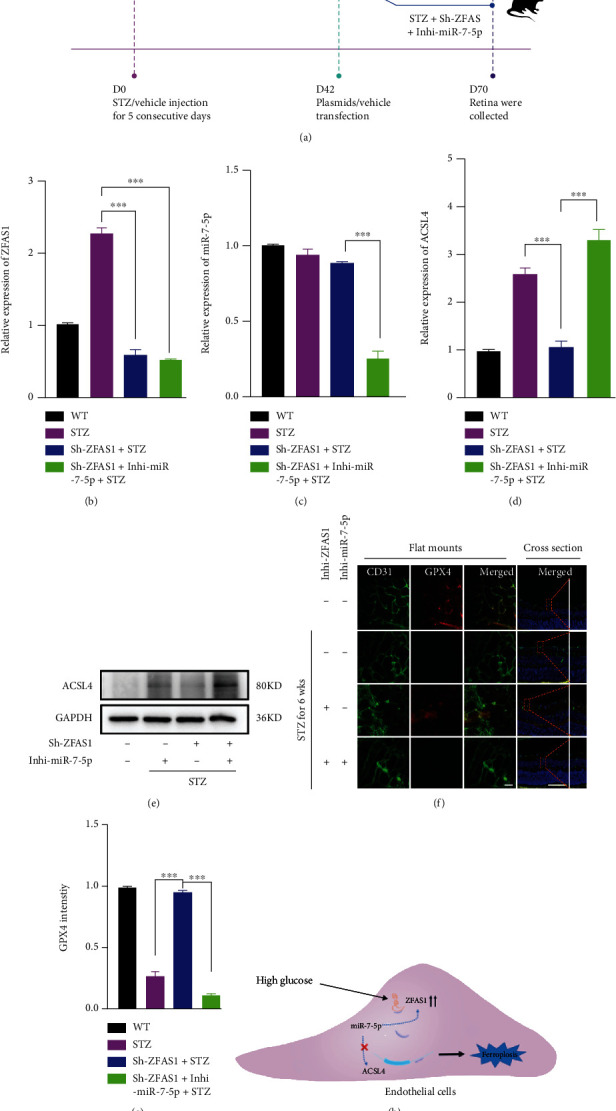
miR-7-5p knockdown partially reverses the functional effect of ZFAS1 knockdown on endothelial ferroptosis in DR mice. (a) Schematic representation of STZ administration. C57BL/6J male mice at 6 weeks old were randomly separated into four experimental groups. Streptozotocin was injected intraperitoneally at a final concentration of 55 mg/kg for 5 consecutive days to induce diabetes, and all diabetic mice were fed with high-fat diet subsequently. Six weeks after the final injection, indicated plasmids (including 100 nM Sh-ZFAS1 and 50 nM Inhi-miR-7-5p) or vehicles were intravitreally injected once a week for four weeks. RT-qPCR validated the expression level of ZFAS1 (b), miR-7-5p (c), and ACSL4 (d). (e) Western blotting analysis validated the ACSL4 protein level. (f) Immunofluorescence assay for GPx4 (labelled red) expression in mouse retinal endothelial cell (labelled green). Scale bar for retinal flat mounts, 50 *μ*m. Scale bar for retinal cross section, 50 *μ*m. (g) Quantification of GPx4 intensity. Data are presented as ± SEM. ^∗∗∗^*P* < 0.001. (h) The summary diagram. ZFAS1 was induced under HG condition. It functioned as a “ceRNA” by sponging miR-7-5p, thus upregulating ACSL4 expression and accelerating ferroptosis-related endothelial dysfunction in DR. All data are presented as ± SEM. ^∗∗∗^*P* < 0.001.

Considering that STZ-induced diabetic mouse is a well-recognized animal model of diabetic retinopathy, we next investigated the effects of ZFAS1/miR-7-5p/ACSL4 axis on endothelial ferroptosis using the STZ mouse model as described before (Figure 5(a)). A total of 48 male mice were assigned to four treatment groups: WT, STZ, Sh-ZFAS1+STZ, and Sh-ZFAS1+Inhi-miR-7-5p+STZ (*n* = 12 in each group). After being isolated from the retinae in four experimental groups using the flow sorting, RECs in each group were subjected to RT-qPCR analysis to have transfection efficiency of Sh-ZFAS1 and Inhi-miR-7-5p evaluated (Figures 5(b) and 5(c)). In line with what we observed *in vitro*, the restraining effect of Sh-ZFAS1 on ACSL4 expression was dramatically abolished after inhi-miR-7-5p treatment, as demonstrated by RT-qPCR and western blotting analysis (Figures 5(d) and 5(e)). Next, retinal flat mounts as well as the cross section revealed that five consecutive days of STZ injection produced classical retinal vascular leakage in six-week-old mice when their retinae were collected at D70 [56]. The downregulation of GP_X_4 expression were observed in all STZ groups, and intravitreally injection of Sh-ZFAS1 markedly rescued the GP_X_4 expression loss, which was further blocked by miR-7-5p inhibition (Figures 5(f) and 5(g)). Taken together, our data indicates that elevated ZFAS1 expression level in STZ mice is responsible for excess oxidative environment in RECs and implies the important modulatory roles played by ZFAS1/miR-7-5p/ACSL4 axis in ferroptosis process.

## 4. Discussion

Among the current treatments of DR, the first and foremost is controlling blood sugar. Multiple evidence has pointed out that changing lifestyle, ameliorating insulin resistance, and repairing damaged *β* islet cell function can effectively delay the occurrence of DR [57–59]. DR is now recognized as a neuro- and vaso-degenerative rather than a microvascular disease, even in early stage when neuron loss is not evident [60, 61]. Recently, the crucial role of ferroptotic cell death has been noted in various neurodegenerative diseases, especially in Alzheimer's disease and Parkinson's disease, while its role in DR is largely unknown [62–65]. Here, we provide the first evidence that in DR, hyperglycemia causes endothelial dysfunction via activating ferroptosis and ZFAS1/miR-7-5p/ACSL4 axis may serve as a key signaling in ferroptosis process.

Reactive oxygen species (ROS) are mainly produced during mitochondrial oxidative metabolism and decisively contribute to multiple cellular signaling pathways, affecting almost all aspects of cellular function including gene expression, proliferation, migration, and cell death [66, 67]. Normally, retinal cells maintain a balance between pro- and antioxidative signaling [68–70]. In diabetes, ROS production in the retina is significantly increased and further exacerbated by the collapse of antioxidant defensive system, including superoxide dismutase (SOD2) and glutathione peroxidase (GP_X_4) [71–74]. In our current work, both total and intercellular ROS in hRECs were elevated, and the expression of GP_X_4 was significantly compromised under hyperglycemia, indicating the highly oxidative environment in endothelial cells under HG. Besides, the ROS level acts as an important promotor of lipid peroxidation-induced ferroptosis [75, 76]. It has been suggested that oxidants may participate in ferroptosis by altering the physical properties of lipid bilayers or increasing membrane curvature and membrane damage through micelle formation [77–79]. In the present study, hyperglycemia-induced ROS accumulation was alleviated by Fer-1 administration. Combined, our data reveals that ferroptosis plays a crucial role in ROS-induced endothelial damage in DR.

ZFAS1 partakes in the pathogenesis of diverse human disorders, including in neurodegenerative disorders, immune responses, and cancer [80–82]. Given that it is mainly localized in cytoplasm, ZFAS1 exerts its effect mostly as a molecular sponge for many miRNAs and ultimately alters the stability and translation of cytoplasmic mRNAs [83, 84]. For instance, it was previously proposed that ZFAS1 acts as an oncogene via sponging miR-329 to facilitate bladder cancer tumorigenesis [85]. Although the targeting site of miR-7-5p on ZFAS1 was validated in nasopharyngeal carcinoma cells [86], very limited efforts have been made in reporting the role, nor the molecular mechanism of ZFAS1 in ocular diseases. Here, we report for the first time that in DR, ZFAS1 expression was upregulated in hRECs. More importantly, our data reveals that high ZFAS1 level induces ROS accumulation and ferroptosis, in agreement with the previous work [47].

Of note, ACSL4, a key positive regulator and biomarker in ferroptosis, was found to be a target gene of ZFAS1/miR-7-5p axis [87]. ACSL4 is required for the production of poly-unsaturated fatty acids required for the execution of ferroptosis. Yuan et al. once reported that ACSL4 depletion by specific shRNA enhances resistance to erastin-induced ferroptosis in cancer cells [88]. By contrast, increase of ACSL4 promotes ferroptosis via activating NF2-YAP signaling [89]. More recently, researchers have indicated the key role of ACSL4 in the onset of DR. The expression level of ACSL4 was found upregulated in retinal pigmental epithelial cells in the early stage of DR, and cells transfected with ACSL4-siRNA were much more resistant to high glucose-induced ferroptosis [90]. In line with the previous report, we noticed that ZFAS1 silencing as well as the miR-7-5p overexpression ameliorated the glucose-induced endothelial ferroptosis phenotypes via downregulating the ACSL4 expression, indicating that the ZFAS1/miR-7-5p/ACSL4 signaling may serve as a promising therapeutic target for DR.

Altogether, we found that the lncRNA ZFAS1 was induced by hyperglycemia in hRECs and proposed that ZFAS1 may exerts its role by competitively binding with miR-7-5p and modulating the expression of its downstream mRNA ACSL4 expression (Figure 5(h)). Our data indicates that ZFAS1 is a major regulator of endothelial dysfunction and could be a new therapeutic target for the DR treatment. Moreover, molecules targeting ZFAS1/miR-7-5p/ACSL4 axis may play an important role in preventing the loss of endothelial cells, capillary occlusion, and the subsequent hypervascularization, which provides a future direction for early intervention of DR.

## 5. Conclusion

In conclusion, we demonstrated that lncRNA ZFAS1 has a ferroptotic effect on the retinal vascular endothelial cells which function as a sponge RNA to miR-7-5p and ultimately regulate ACSL4 expression. Our data strongly suggests that lncRNA ZFAS1 is a key contributor to the development of DR.

## Data Availability

The original contributions presented in the study are included in the article material; further inquiries can be directed to the corresponding author.

## Supplementary Materials

Supplementary Materials Supplementary Figure 1: identification of primarily cultured hRECs. Supplementary Figure 2: the effects of GPx4 overexpression on ZFAS1/miR-7-5p/ACSL4 axis. Supplementary Table 1: RNA sequences and the primer sequences for RT-qPCR. Supplementary Table 2: a total of 108 dysregulated lncRNAs were identified between the endothelial cells isolated from nine fibrovascular membrane (FVM) samples and four control retinal samples without diabetes diagnosis. Supplementary Table 3: a total of 69 miRNAs were identified to interact with ZFAS1 using starBase database. Supplementary Table 4: a total of 54 potential target genes of miR-7-5p were predicted using predictive datasets miRDB, DIANA, miRmap, and PicTar.
