# Supplementary material for: lncRNA ZFAS1 Positively Facilitates Endothelial Ferroptosis via miR-7-5p/ACSL4 Axis in Diabetic Retinopathy
Source: Oxid Med Cell Longev. 2022 Aug 31;2022:9004738. doi: 10.1155/2022/9004738 (PMC9453005; doi:10.1155/2022/9004738)
Supplement: Supplementary Materials — Supplementary Figure 1: identification of primarily cultured hRECs. Supplementary Figure 2: the effects of GPx4 overexpression on ZFAS1/miR-7-5p/ACSL4 axis. Supplementary Table 1: RNA sequences and the primer sequences for RT-qPCR. Supplementary Table 2: a total of 108 dysregulated lncRNAs were identified between the endothelial cells isolated from nine fibrovascular membrane (FVM) samples and four control retinal samples without diabetes diagnosis. Supplementary Table 3: a total of 69 miRNAs were identified to interact with ZFAS1 using starBase database. Supplementary Table 4: a total of 54 potential target genes of miR-7-5p were predicted using predictive datasets miRDB, DIANA, miRmap, and PicTar. [file 9004738.f1.pdf]

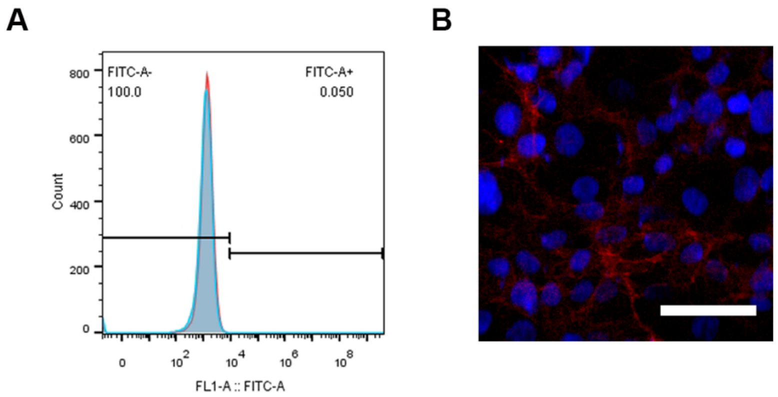

Supplementary Figure 1 Identification of primarily cultured hRECs.

(A) Flow cytometric analysis of characteristic cell marker CD31. Red curves represent isotype controls for PE dye and blue curves represent measured endothelial marker CD31. (B) Primarily cultured cells at passage 1 stained positively for the endothelial cell marker CD31 (scale bar = 40 μm).

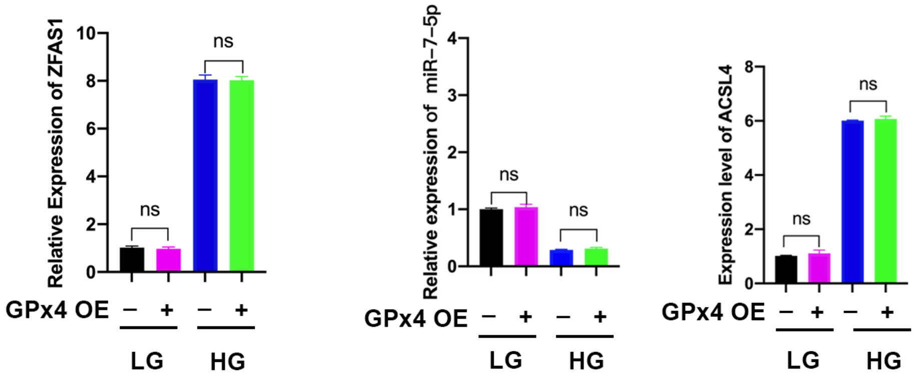

Supplementary Figure 2 The effects of GPx4 overexpression on ZFAS1/miR-7-5p/ACSL4 axis.

RT-qPCR results demonstrated that no detectable alteration was observed in ZFAS1, miR-7-5p, and ACSL4 expression level after GPx4 overexpression (GPx4 OE). GAPDH and miR-39-3p served as endogenous and exogeous normalization for ACSL4 and miR-7-5p detection, respectively. ns means not significant.

Supplementary Table 1 RNA sequences and the primer sequences for RT-qPCR

**Oligonucleotide**	**Forward**	**Reverse**
mimic-NC	5′UUCUCCGAACGUGUCACGUTT-3′	
miR-7-5p mimic	5′UGGAAGACUAGUGAU UUUGUUGU-3′	5′AACAAAAUCACUAGUCUUCCA UU-3′
si-ACSL4	5’-TAGATATCAGTTGTGTTAA-3’	

**Primer**	**Forward**	**Reverse**
ZFAS1	5′ACGTGCAGACATCTACAACCT3 ′	5′TACTTCCAACACCCGCAT-3′
ACSL4	5′CATCCCTGGAGCAGATACTCT-3′	5′TCACTTAGGATTTCCCTGGTCC-3′
GAPDH	5′AGGTGAAGGTCGGAGTCAACG –3′	5′AGGGGTCATTGATGGCAACA-3′
U6	5′CTCGCTTCGGCAGCACATATAC T-3′	5′ACGCTTCACGAATTTGCGTGTC-3′

Supplementary Table 2 A total 108 dysregulated lncRNAs were identified between the $CD31^+$ endothelial cells isolated from nine fibrovascular membranes (FVM) samples and four control retinal samples without diabetes diagnosis using a differential gene expression analysis.

Geen id	logFC	AveExpr	t	P.Value	B

DLGAP1-AS3	-1.4710454	0.32849254	-10.757025	1.33E-07	7.69655376
FAM155A-IT1	-2.9211402	0.78065509	-7.9044537	3.72E-06	4.74588383
DIO3OS	-4.4987755	1.19044822	-7.3215001	8.17E-06	4.01606643
LINC01267	-4.3167197	1.09421478	-6.6779134	2.04E-05	3.15264547
LEMD1-AS1	-1.4600016	0.44365662	-6.5273842	2.55E-05	2.94163319
ENTPD3-AS1	-1.2735467	0.37366433	-6.3628158	3.26E-05	2.70692936
LINC01559	-3.71664	0.96429112	-6.2094107	4.12E-05	2.48434696
PWRN1	-1.926368	0.39854217	-5.9447623	6.21E-05	2.09169043
LINC01511	-1.4352726	0.37756622	-5.9377284	6.28E-05	2.08110433
PLCE1-AS2	-4.8388368	1.6898932	-5.5988212	0.00010762	1.56185195
CLIP1-AS1	-2.5400884	0.81229483	-5.4658787	0.00013356	1.35327261

LINC01231	-1.7238205	0.55908245	-5.4423255	0.000138805	1.31603394
FRY-AS1	-3.3454689	1.16242564	-5.4386667	0.000139639	1.31024142
LINC00710	-1.4622803	0.40781713	-5.1257507	0.000234702	0.80730143
LINC01555	-4.4836692	1.64790877	-4.9683099	0.000306344	0.54872097
LINC00507	-1.6122581	0.3915245	-4.8958007	0.000346726	0.42842334
LINC01091	-1.1770119	0.4608682	-4.8309834	0.000387543	0.32025547
LHFPL3-AS2	-2.5479843	0.67044479	-4.6363714	0.000543118	-0.007996
CATIP-AS2	-2.7391397	0.69985332	-4.5979709	0.000580858	-0.0733644
SPATA41	-2.220901	0.72328936	-4.5259727	0.000659161	-0.1964381
ZFAS1	3.75670016	4.26267847	4.46863673	0.000729342	-0.2949156
ZNF252P-AS1	-1.9932482	0.58966903	-4.4054046	0.00081582	-0.4039868

TGFB2-OT1	-1.7759999	0.6270409	-4.1001359	0.001410604	-0.9369247
LINC01132	-3.5890891	1.27942644	-4.054843	0.001531344	-1.016815
MIF-AS1	2.92044245	3.10096112	4.02268047	0.001623517	-1.0736603
COLCA1	-1.7823379	0.74214159	-3.9360194	0.001901424	-1.227278
LINC00994	-3.4418831	1.32645915	-3.7564406	0.002643953	-1.5474482
LINC01289	-4.0875048	1.77950551	-3.7273242	0.002789858	-1.5995609
CASC20	-3.5954107	1.38027106	-3.690425	0.002986677	-1.6656721
C8orf49	-4.6300025	2.4551697	-3.6641287	0.00313556	-1.7128301
TCL6	-3.8442528	1.54240611	-3.6413913	0.003270399	-1.753633
LINC01360	-2.9911909	0.92518848	-3.4846101	0.004376249	-2.0355345
MIR22HG	3.9007892	3.94647777	3.36044039	0.005517306	-2.2592219

WDR11-AS1	-3.2199691	1.70159224	-3.3523134	0.005601754	-2.2738673
ERVK13-1	2.30362055	4.1505639	3.33131008	0.005826108	-2.3117165
KRBOX1-AS1	-1.7762186	0.70401075	-3.3146805	0.006010172	-2.3416832
SORCS3-AS1	-1.6284024	0.74873236	-3.2931348	0.006257419	-2.3805065
LINC00870	-3.5484501	1.60596279	-3.2265699	0.007088062	-2.5004138
WDFY3-AS2	-1.5824012	0.65104239	-3.2185327	0.007195603	-2.5148863
LINC01520	-1.0593642	0.30147837	-3.0941926	0.009084613	-2.7385254
LINC00471	-2.0278403	0.96270184	-3.0698271	0.009509416	-2.7822735
SLC39A12-AS1	-1.853533	0.50693687	-3.0429587	0.010000938	-2.8304781
LINC00707	-3.9863704	2.78169011	-3.0199029	0.01044291	-2.8718081
GAS5	3.67090603	3.96884949	2.99394702	0.010963852	-2.9182953

LINC00930	-1.7437713	0.88717996	-2.9827235	0.011197064	-2.9383821
LINC00691	-1.2761787	0.66753253	-2.9793645	0.011267816	-2.9443919
LINC00299	-2.4687919	1.4208337	-2.9760496	0.011338076	-2.950322
TMCC1-AS1	-3.4730649	1.82214051	-2.9253233	0.012469289	-3.0409593
NAV2-AS5	-4.0061997	2.55890654	-2.8600782	0.014090593	-3.1572038
DNAJC9-AS1	-1.692498	0.74530722	-2.8564551	0.014186513	-3.1636466
LINC00158	-2.2446696	1.30936031	-2.8494676	0.014373337	-3.1760685
LINC00901	-2.0512018	1.14524051	-2.8396262	0.014640603	-3.193555
CEBPB-AS1	-2.8502869	1.59329985	-2.8372493	0.014705886	-3.1977767
C8orf31	-2.7555551	0.7931688	-2.8048286	0.015625631	-3.2552983
LINC00462	-1.3924286	0.35271905	-2.8017637	0.015715465	-3.2607298

LINC00550	-2.5160656	1.66399517	-2.7893621	0.016084203	-3.2826962
LINC00606	-3.2198475	1.69891621	-2.7792604	0.016390856	-3.3005751
LINC01299	-3.4557692	2.51204606	-2.7444078	0.017493774	-3.3621609
LINC00652	-2.0189293	0.51893776	-2.7358769	0.017774684	-3.3772111
LINC01516	-1.7616249	0.92262286	-2.729095	0.018001163	-3.3891685
LINC-PINT	3.20856617	2.94040549	2.7132037	0.018543025	-3.4171622
LINC01204	-1.696483	0.82943962	-2.7091311	0.018684451	-3.4243306
EGFLAM-AS2	-2.8778582	1.82633626	-2.6927913	0.01926261	-3.4530672
SNHG6	2.72830295	4.96389472	2.69213139	0.019286325	-3.4542269
LINC00917	-2.9987067	0.839286	-2.6649083	0.020289999	-3.5020125
HCG11	-3.3183918	3.06540302	-2.6535198	0.020724899	-3.5219689

LINC00927	-3.2509643	1.75451787	-2.6198416	0.02206507	-3.5808622
DSG2-AS1	-1.6797904	0.64172906	-2.594522	0.023128019	-3.6250125
LINC01561	-2.7690085	0.78478968	-2.5924672	0.023216444	-3.6285907
LINC01247	-4.0670694	2.58861767	-2.5809988	0.023716043	-3.6485473
CACNA1C-AS4	-3.7199238	2.71771959	-2.5779088	0.023852436	-3.6539202
ERICH3-AS1	-3.0777581	1.9750823	-2.5764865	0.023915469	-3.6563927
ENTPD1-AS1	-3.5424245	2.78354374	-2.5688178	0.024258154	-3.6697177
LINC00967	-4.1361636	2.61677851	-2.5668475	0.024346969	-3.6731395
GTF3C2-AS1	-2.2894118	0.93493783	-2.5536367	0.024950692	-3.6960638
THUMPD3-AS1	3.57162659	4.18358453	2.53709061	0.025727402	-3.7247292
LINC00485	-2.1483497	1.27809691	-2.5293725	0.026097695	-3.7380824

GPR1-AS	-2.162699	1.31894136	-2.5207087	0.026519523	-3.7530577
DDX11-AS1	-1.3521424	0.52816121	-2.5164176	0.026730893	-3.7604692
LARS2-AS1	-1.7354155	0.94717719	-2.5154218	0.026780176	-3.7621886
LINC00426	-1.9647512	0.96796443	-2.5024741	0.027429058	-3.7845266
TUB-AS1	-3.3760009	2.04821534	-2.4973863	0.027688187	-3.793295
LINC00701	-3.5311339	3.67608606	-2.4918689	0.027971877	-3.8027978
LINC00269	-1.54763	0.5063354	-2.4817521	0.028499382	-3.8202056
NRSN2-AS1	-1.3433054	0.45008381	-2.4664604	0.029315025	-3.8464763
LINC01266	-2.9083933	2.42264595	-2.4408585	0.030731354	-3.8903456
SNAP25-AS1	-1.8254407	0.69221126	-2.3872799	0.033911959	-3.9816688
LINC01550	-1.3319192	0.54597387	-2.366477	0.035230274	-4.0169415

CASC17	-2.5714905	1.23600414	-2.3462516	0.03655902	-4.0511311
LINC00271	-2.0483918	1.31128128	-2.3244866	0.038042462	-4.0878055
HCG18	1.32947769	1.75037925	2.31586476	0.038645873	-4.102299
CLSTN2-AS1	-2.5807952	1.43979741	-2.3137852	0.038792779	-4.1057919
NAV2-AS4	-2.1910735	0.75067247	-2.3060884	0.039341138	-4.1187092
PROSER2-AS1	-2.3186002	1.43534106	-2.250357	0.043537904	-4.2117522
LINC00842	-3.1482492	2.67402138	-2.2421609	0.04418988	-4.2253606
FGD5-AS1	2.09601714	4.26070488	2.23637428	0.044655758	-4.2349565
IGF2BP2-AS1	-1.2642556	0.72709584	-2.2349325	0.04477256	-4.237346
PTCSC3	-2.0227407	1.27536807	-2.2234661	0.045711817	-4.2563263
PTPRD-AS2	-2.8191703	1.59303051	-2.2224004	0.045800061	-4.2580885

FAM225A	2.46007457	2.18305474	2.21591476	0.046340541	-4.2688045
SNHG16	2.61728865	5.51067429	2.21051176	0.046795406	-4.2777221
SMAD5-AS1	-1.6294541	0.61056571	-2.2063384	0.047149636	-4.284604
LINC01206	-4.7424641	3.95644796	-2.1878765	0.048747287	-4.3149834
LINC00840	-2.5988943	1.84129049	-2.1871159	0.048814191	-4.3162327
TMEM72-AS1	-1.0542429	0.55922908	-2.1867444	0.048846902	-4.3168429
C3orf35	-3.3280128	2.66326454	-2.178812	0.049550279	-4.3298601
NUTM2B-AS1	2.3553037	3.76727381	2.17574485	0.049824804	-4.3348881

Supplementary Table 3 A total of 69 miRNAs were identified to interact with ZFAS1 using starBase database.

miRNAid	miRNAname	geneID	start	end
MIMAT0000690	hsa-miR-296-5p	ENSG00000177410	47894715	47894724

MIMAT0019699	hsa-miR-4640-5p	ENSG00000177410	47894729	47894751
MIMAT0019845	hsa-miR-4726-5p	ENSG00000177410	47894729	47894751
MIMAT0000758	hsa-miR-135b-5p	ENSG00000177410	47895147	47895174
MIMAT0000428	hsa-miR-135a-5p	ENSG00000177410	47895149	47895174
MIMAT0003293	hsa-miR-624-5p	ENSG00000177410	47895192	47895212
MIMAT0021020	hsa-miR-5000-3p	ENSG00000177410	47895225	47895251
MIMAT0005877	hsa-miR-1286	ENSG00000177410	47895227	47895252
MIMAT0018952	hsa-miR-4436a	ENSG00000177410	47895229	47895251
MIMAT0003312	hsa-miR-642a-5p	ENSG00000177410	47895660	47895684
MIMAT0002814	hsa-miR-432-5p	ENSG00000177410	47895684	47895704
MIMAT0004814	hsa-miR-654-3p	ENSG00000177410	47895701	47895722

MIMAT0004814	hsa-miR-654-3p	ENSG00000177410	47895711	47895722
MIMAT0018199	hsa-miR-3924	ENSG00000177410	47897079	47897100
MIMAT0000095	hsa-miR-96-5p	ENSG00000177410	47897098	47897122
MIMAT0005796	hsa-miR-1271-5p	ENSG00000177410	47897098	47897122
MIMAT0000252	hsa-miR-7-5p	ENSG00000177410	47897110	47897132
MIMAT0004977	hsa-miR-934	ENSG00000177410	47897123	47897146
MIMAT0002833	hsa-miR-520a-5p	ENSG00000177410	47897138	47897158
MIMAT0002838	hsa-miR-525-5p	ENSG00000177410	47897138	47897158
MIMAT0019700	hsa-miR-4640-3p	ENSG00000177410	47897143	47897164
MIMAT0003339	hsa-miR-421	ENSG00000177410	47897246	47897272
MIMAT0000459	hsa-miR-193a-3p	ENSG00000177410	47897271	47897292

MIMAT0002819	hsa-miR-193b-3p	ENSG00000177410	47897273	47897292
MIMAT0005919	hsa-miR-548o-3p	ENSG00000177410	47897274	47897296
MIMAT0005795	hsa-miR-1323	ENSG00000177410	47897275	47897296
MIMAT0019801	hsa-miR-4703-5p	ENSG00000177410	47897340	47897365
MIMAT0018358	hsa-miR-3942-5p	ENSG00000177410	47897344	47897365
MIMAT0014983	hsa-miR-3121-3p	ENSG00000177410	47897373	47897394
MIMAT0000451	hsa-miR-150-5p	ENSG00000177410	47897429	47897448
MIMAT0002834	hsa-miR-520a-3p	ENSG00000177410	47897666	47897691
MIMAT0000724	hsa-miR-372-3p	ENSG00000177410	47897667	47897691
MIMAT0000684	hsa-miR-302a-3p	ENSG00000177410	47897669	47897691
MIMAT0000715	hsa-miR-302b-3p	ENSG00000177410	47897669	47897691

MIMAT0000717	hsa-miR-302c-3p	ENSG00000177410	47897669	47897691
MIMAT0000718	hsa-miR-302d-3p	ENSG00000177410	47897669	47897691
MIMAT0000726	hsa-miR-373-3p	ENSG00000177410	47897670	47897691
MIMAT0002856	hsa-miR-520d-3p	ENSG00000177410	47897670	47897691
MIMAT0002825	hsa-miR-520e	ENSG00000177410	47897671	47897691
MIMAT0002846	hsa-miR-520c-3p	ENSG00000177410	47897671	47897691
MIMAT0002843	hsa-miR-520b	ENSG00000177410	47897672	47897691
MIMAT0005931	hsa-miR-302e	ENSG00000177410	47897675	47897691
MIMAT0002838	hsa-miR-525-5p	ENSG00000177410	47905597	47905617
MIMAT0002833	hsa-miR-520a-5p	ENSG00000177410	47905599	47905617
MIMAT0027571	hsa-miR-6835-3p	ENSG00000177410	47905619	47905642

MIMAT0004797	hsa-miR-582-3p	ENSG00000177410	47905651	47905672
MIMAT0018199	hsa-miR-3924	ENSG00000177410	47905672	47905694
MIMAT0002882	hsa-miR-510-5p	ENSG00000177410	47905679	47905702
MIMAT0010251	hsa-miR-449c-5p	ENSG00000177410	47905736	47905762
MIMAT0000685	hsa-miR-34b-5p	ENSG00000177410	47905738	47905762
MIMAT0013517	hsa-miR-2682-5p	ENSG00000177410	47905738	47905762
MIMAT0015050	hsa-miR-323b-3p	ENSG00000177410	47907425	47907441
MIMAT0002823	hsa-miR-512-3p	ENSG00000177410	47912146	47912171
MIMAT0000684	hsa-miR-302a-3p	ENSG00000177410	47912146	47912172
MIMAT0000715	hsa-miR-302b-3p	ENSG00000177410	47912146	47912172
MIMAT0000717	hsa-miR-302c-3p	ENSG00000177410	47912146	47912172

MIMAT0000718	hsa-miR-302d-3p	ENSG00000177410	47912146	47912172
MIMAT0000724	hsa-miR-372-3p	ENSG00000177410	47912146	47912172
MIMAT0025464	hsa-miR-6504-5p	ENSG00000177410	47912147	47912167
MIMAT0019864	hsa-miR-3064-5p	ENSG00000177410	47912148	47912167
MIMAT0002834	hsa-miR-520a-3p	ENSG00000177410	47912151	47912172
MIMAT0000726	hsa-miR-373-3p	ENSG00000177410	47912152	47912172
MIMAT0002825	hsa-miR-520e	ENSG00000177410	47912152	47912172
MIMAT0002846	hsa-miR-520c-3p	ENSG00000177410	47912152	47912172
MIMAT0002856	hsa-miR-520d-3p	ENSG00000177410	47912152	47912172
MIMAT0002843	hsa-miR-520b	ENSG00000177410	47912153	47912172
MIMAT0005931	hsa-miR-302e	ENSG00000177410	47912157	47912172

MIMAT0025475	hsa-miR-6509-3p	ENSG00000177410	47912170	47912191
MIMAT0004799	hsa-miR-589-5p	ENSG00000177410	47912189	47912212

Supplementary Table 4 A total of 54 potential target genes of miR-7-5p were predicted using predictive datasets miRDB, DIANA, miRmap, and PicTar.

ABCG4	CCNT2	HELLS	PDE4D	SCAMP5
ACSL4	CGGBP1	HPCAL4	PFN2	SEMA4C
ANKFY1	CKAP4	IRS2	PLCB1	SERP1
ANKRD12	CNN3	KLF12	POGK	SLC4A7
ARF4	CNOT8	KLF4	POLE4	SMARCD1
ARID4A	CSMD3	KPNA1	PPARGC1A	SNCA
ATP2B2	DACH1	LEMD3	PSME3	SPATA2
ATRX	EIF4EBP2	NR4A3	RAF1	TFRC
BMPR2	ESRRG	NXT2	RB1	VDAC1
CALU	FLRT2	OGT	RNF141	ZNF395
CAPZA1	GLI3	OSBPL11	RSBN1	
